# Association between fat mass through adolescence and arterial stiffness: a population-based study from The Avon Longitudinal Study of Parents and Children

**DOI:** 10.1016/S2352-4642(19)30105-1

**Published:** 2019-07

**Authors:** Frida Dangardt, Marietta Charakida, Georgios Georgiopoulos, Scott T Chiesa, Alicja Rapala, Kaitlin H Wade, Alun D Hughes, Nicholas J Timpson, Konstantinos Pateras, Nick Finer, Naveed Sattar, George Davey Smith, Debbie A Lawlor, John E Deanfield

**Affiliations:** a Vascular Physiology Unit, Institute of Cardiovascular Science, University College London, London, UK; b Department of Population Science and Experimental Medicine, UCL Institute of Cardiovascular Science, University College London, London, UK; c MRC Unit for Lifelong Health and Ageing at UCL, University College London, London, UK; d Department of Paediatric Clinical Physiology, The Queen Silvia Children's Hospital, Sahlgrenska Academy and University Hospital, Gothenburg, Sweden; e School of Biomedical Engineering and Imaging Sciences, King's College London, UK; f Department of Clinical Therapeutics, National and Kapodistrian University of Athens, Athens, Greece; g MRC Integrative Epidemiology Unit, Population Health Sciences, Bristol Medical School, University of Bristol, Bristol, UK; h Department of Biostatistics and Research Support, Julius Center for Health Sciences and Primary Care, University Medical Center Utrecht, Utrecht, The Netherlands; i Institute of Cardiovascular and Medical Sciences, British Heart Foundation Glasgow, Cardiovascular Research Centre, University of Glasgow, Glasgow, UK

## Abstract

**Background:**

The link between adiposity, metabolic abnormalities, and arterial disease progression in children and adolescents remains poorly defined. We aimed to assess whether persistent high adiposity levels are associated with increased arterial stiffness in adolescence and any mediation effects by common metabolic risk factors.

**Methods:**

We included participants from the Avon Longitudinal Study of Parents and Children (ALSPAC) who had detailed adiposity measurements between the ages 9–17 years and arterial stiffness (carotid to femoral pulse wave velocity [PWV]) measured at age 17 years. Body-mass index (BMI) and waist-to-height ratio were calculated from weight, height, and waist circumference measurements whereas fat mass was assessed using repeated dual-energy x-ray absorptiometry (DEXA) scans. We used total and trunk fat mass indices (FMIs) to classify participants as normal (<75th percentile) or high (>75th percentile) FMI. We classified participants as being metabolically unhealthy if they had three or more of the following risk factors: high levels of systolic blood pressure, triglycerides, or glucose (all >75th percentile) or low levels of high-density lipoprotein (<25th percentile). We used multivariable linear regression analysis to assess the relationship between PWV and exposure to adiposity, and tested for linear trend of PVW levels across ordinal groups. We used latent class growth mixture modelling analysis to assess the effect of longitudinal changes in adiposity indices through adolescence on arterial stiffness.

**Findings:**

We studied 3423 participants (1866 [54·5%] female and 1557 [45·5%] male). Total fat mass was positively associated with PWV at age 17 years (0·004 m/s per kg, 95% CI 0·001–0·006; p=0·0081). Persistently high total FMI and trunk FMI between ages 9 and 17 years were related to greater PWV (0·15 m/s per kg/m^2^, 0·05–0·24; p=0·0044 and 0·15 m/s per kg/m^2^, 0·06–0·25; p=0·0021) compared with lower FMI. Metabolic abnormalities amplified the adverse effect of high total FMI on arterial stiffness (PWV 6·0 m/s [95% CI 5·9–6·0] for metabolically healthy participants and 6·2 m/s [5·9–6·4] for metabolically unhealthy participants). Participants who restored normal total FMI in adolescence (PWV 5·8 m/s [5·7–5·9] for metabolically healthy and 5·9 m/s [5·6–6·1] for metabolically unhealthy) had comparable PWV to those who had normal FMI throughout (5·7 m/s [5·7–5·8] for metabolically healthy and 5·9 m/s [5·8–5·9] for metabolically unhealthy).

**Interpretation:**

Persistently high fat mass during adolescence was associated with greater arterial stiffness and was further aggravated by an unfavourable metabolic profile. Reverting to normal FMI in adolescence was associated with normal PWV, suggesting adolescence as an important period for interventions to tackle obesity in the young to maximise long-term vascular health.

**Funding:**

UK Medical Research Council, Wellcome Trust, British Heart Foundation, and AFA Insurances

## Introduction

Over the past four decades, there has been an alarming increase in the prevalence and incidence of obesity in both developed and developing countries,^1^ with the fastest rises in adiposity levels reported in children, adolescents, and young adults. This increase at younger ages represents a growing threat to worldwide public health because obesity in childhood tracks to adulthood and is associated with a disturbed cardiometabolic profile and accelerated progression of atherosclerotic disease in adulthood.^2^, ^3^, ^4^ Although the association between adiposity and cardiovascular disease in adults is well described, the link between adiposity and arterial disease progression in children and adolescents remains unclear.

Our group has previously shown that children with adiposity had a higher heart rate, greater resting and reactive hyperaemic blood flow, and larger arterial diameters compared with children with normal weight.^5^, ^6^, ^7^, ^8^ Children with adiposity had a greater endothelial function and less arterial stiffness than did children of a normal weight, suggesting an adaptive hyperaemic state in response to pre-pubertal adiposity. However, whether similar adaptive responses, which are not necessarily unhealthy at this stage, persist in adolescence remains to be established. Because of rapid changes in body composition during adolescence, direct and repeated measurements of fat are needed to decipher the independent effect of growth from that of adiposity on vascular health.

**Research in context**

**Evidence before this study**

Adiposity in children and adolescents represents a major public health challenge and a driver of both diabetes and cardiovascular disease in childhood and in later life. There has been controversy about the importance of fat phenotype and associated metabolic disturbances on the initiation and progression of early arterial disease. We searched Google Scholar and PubMed from inception to Dec 18, 2018, for references from studies on longitudinal adiposity, associated with measurement of vascular stiffness and metabolic health in a large population of children. The search terms we used included “longitudinal adiposity OR obesity OR DXA”, “children OR adolescents”, “vascular OR arterial stiffness” and “metabolic”. No previous studies in children or adolescents using serial dual-energy x-ray absorptiometry measurements for longitudinal assessment of adiposity throughout adolescence as well as measurements of metabolic health and arterial stiffness were found in our search.

**Added value of this study**

We showed that participants with persistent adiposity (measured as increased fat mass) between 9 and 17 years of age had worse arterial stiffness at age 17 years and that restoration of a normal fat mass was associated with arterial stiffness levels that were comparable to those who had normal fat mass throughout childhood and adolescence. The association between high fat mass and greater arterial stiffness was further aggravated when metabolic abnormalities such as dyslipidaemia and increased blood pressure coexisted with increased adiposity.

**Implications of all the available evidence**

Combined with previous findings, the results of our study have important implications for preventive approaches, which need to start in early life and have the potential to influence favourably the development of arterial disease. Future research needs to not only understand the control of fat mass development in childhood and adolescence, but also determine effective interventions to prevent and treat childhood overweight and obesity.

In the Avon Longitudinal Study of Parents and Children (ALSPAC), we used dual-energy x-ray absorptiometry (DEXA) and other proxy estimates of adiposity such as body-mass index (BMI) and waist-to-height ratio to characterise persistent adiposity from childhood to young adulthood and its effect on arterial stiffness at 17 years of age. This longitudinal assessment provided us with the opportunity to construct trajectories of adiposity and to address the specific questions of whether persistent high adiposity levels are associated with increased arterial stiffness in adolescence and, if so, whether this effect is mediated by the presence of other metabolic risk factors commonly seen in individuals with adiposity.

## Methods

### Study design and participants

ALSPAC is a prospective birth cohort study investigating factors that influence normal childhood development and growth. The cohort and study design have been described in detail previously and are available on the ALSPAC website.^9^, ^10^ For our analysis, we included all participants who had detailed adiposity measurements using repeated DEXA scans at ages 9 and 17 years and arterial stiffness measured at age 17 years. Ethical approval for all aspects of this study was obtained from the ALSPAC Ethics and Law Committee and the Local Research Ethics Committee and conformed to the Declaration of Helsinki.

### Procedures

To characterise adiposity, weight, height, and waist circumference were measured at 2-year intervals from ages 9 to 17 years, from which BMI and waist-to-height ratio were calculated.^5^ Fat mass was assessed using DEXA at ages 9, 11, 13, 15, and 17 years. The reproducibility of DEXA measures in our cohort has been previously reported,^11^ and the repeatability coefficient was 0·5 kg for total body mass. Total and trunk fat mass indices (FMIs) were calculated by dividing total and trunk fat mass by squared height, whereas the fat-free mass index was generated by dividing fat-free mass by squared height. Participants were classified as high FMI if total sex-and height-adjusted FMI was greater than the 75th percentile of the dataset. Detailed information about the methods used is found in the appendix.

Vascular phenotype was determined when participants were 17 years old: pressure waveforms were obtained using the Vicorder device (Skidmore Medical, Bristol, UK) at carotid and femoral artery level and pulse wave velocity (PWV) was calculated using an inbuilt cross-correlation algorithm previously validated for use in adolescents.^12^ All measurements were taken independently by one of two trained vascular technicians (one of whom was AR; inter-observer mean difference 0·2 m/s, SD 0·1).

Blood pressure was recorded at ages 9 and 17 years. Non-fasting blood samples were taken using standard procedures at age 9 years, whereas overnight fasting samples were collected for analysis at age 17 years. Plasma lipids (total cholesterol, triglycerides, and high-density lipoprotein [HDL] cholesterol) were measured by modification of the standard Lipid Research Clinics protocol using enzymatic reagents for lipid determination. All assays were considered reliable, with small coefficients of variation of less than 5%. Non-HDL cholesterol was calculated as total cholesterol minus HDL cholesterol. The amount of weekly vigorous physical activity was assessed by accelerometer at ages 9 and 15 years. Further details regarding cardiovascular risk factor assessment can be found in the appendix.

Similarly to the modified National Cholesterol Education Program and International Diabetes Federation definitions of paediatric metabolic syndrome,^13^ participants were classified as metabolically unhealthy at either 9 or 17 years of age if they had three or more of the following risk factors: systolic blood pressure higher than the 75th percentile, HDL lower than the 25th percentile, triglycerides higher than the 75th percentile, and glucose higher than the 75th percentile.^13^

### Statistical analysis

All normally distributed variables are expressed as mean (SD). Normal distribution of parameters that were used as dependent variables in statistical tests was assessed by histograms and quantile–quantile plots. Partial correlations between adiposity measurements were assessed with Spearman's correlation coefficient. Unadjusted comparisons of variables between male and female participants were done using linear regression, independent-samples Student's *t* test, Mann-Whitney *U* test, or χ^2^ test.

We used multivariable linear regression analysis to assess the relationship between PWV and exposure to adiposity. In the primary complete-case analysis (ie, the core model), we included the following covariates: sex, socioeconomic status, low-density lipoprotein (LDL) cholesterol, smoking, systolic blood pressure, C-reactive protein, birthweight, and BMI Z score at 17 years. Total fat mass was adjusted for height where applicable. Subsequently, we did a secondary analysis (ie, the expanded multivariable model) that controlled for the following additional confounders or mediators: accelerometer data for physical activity at age 15 years, parental and participant's smoking, and puberty status (Tanner stage; appendix). In this analysis, we replaced all missing data by multiple imputation using the Markov chain Monte Carlo method (appendix). We used observed and imputed data on exposure cardiometabolic variables to derive combined categories of metabolic health (metabolically healthy *vs* metabolically unhealthy) and total FMI status on the two occasions with maximum time difference (ages 9 and 17 years). A test for linear trend (ie, levels of PWV across ordinal groups) was done across prespecified categories of interest, based on specific combinations of baseline and changes in fat mass and metabolic status, after regression models. A positive test for trend suggests a linear association (increase or decrease) between the dependent variable (PWV) and the levels of the ordinal independent variable; in other words, the effect of combined fat exposure and metabolic derangements on PWV will linearly increase per transition from lower to higher category, irrespectively of which category a child is classified in. To assess the effect of longitudinal changes in adiposity indices through adolescence on arterial stiffness, we used latent class growth (LCG) mixture modelling analysis.^14^ Multiple measurements of total fat, trunk fat, lean mass, and total body mass were indexed for height changes (ie, height squared). The LCG models contained random intercept and slope variances to account for between-subject heterogeneity in longitudinal changes in obesity measurements while the linear, quadratic, and cubic specifications for the within-subject response of these variables as a function of increasing age were evaluated (appendix).

We used Stata, version 13.1, for statistical analysis and the lcmm package in RStudio (version 1.1.414) in LCG mixture modelling analysis. All tests were two sided. A priori, we planned to draw conclusions on the basis of effect estimates and their CIs, rather than statistical tests using an arbitrary p value cutoff. For example, given two effects with the same point estimate—one with narrow CIs, the other with wider CIs that could even include the null—we describe both as showing the same effect but note that one is more imprecisely estimated and should be treated with more caution until replicated in a larger sample.

### Role of the funding source

The funders had no role in the design and conduct of the study; collection, management, analysis, and interpretation of data; and preparation, review, or approval of the manuscript. The corresponding author had full access to all of the data and the final responsibility to submit for publication.

## Results

We included 3423 participants in this study, of whom 1557 (45·5%) were male and 1866 (54·5%) were female (table 1). Of these participants, 3046 (89%) had DEXA and height measurements at ages 9 and 17 years. 1990 (65%) participants had normal FMI (ie, <75th percentile) in both periods, 294 (10%) initially had normal FMI but increased to high FMI at age 17 years, 288 (9%) had high FMI at age 9 years but normal FMI at age 17 years, and 474 (16%) had consistently high FMI. The association between FMI status and cardiometabolic parameters at ages 9 and 17 years is shown in table 2.

**Table 1:** Patient characteristics at ages 9 years and 17 years

	**Age 9 years**			**Age 17 years**			p value for change*
	Female (n=1866)	Male (n=1557)	p value	Female (n=1866)	Male (n=1557)	p value	
Weight, kg	38·0 (8·1)	37·3 (7·4)	0·0064	61·9 (11·6)	71·1 (11·9)	<0·0001	<0·0001
Height, cm	140 (7)	140 (6)	0·81	166 (6)	179 (7)	<0·0001	<0·0001
BMI, kg/m^2^	18·2 (2·9)	17·8 (2·7)	0·00037	22·6 (3·9)	22·2 (3·4)	0·0014	0·60
Waist-to-height ratio	0·44 (0·42–0·42)	0·44 (0·42–0·47)	0·51	0·45 (0·43–0·49)	0·43 (0·41–0·45)	<0·0001	<0·0001
Total fat mass, kg	8·4 (5·9–11·7)	5·7 (4·0–8·9)	<0·0001	19·2 (14·8–25·1)	10·2 (6·9–16·6)	<0·0001	<0·0001
Trunk fat mass, kg	3·2 (2·1–4·9)	2·0 (1·4–3·4)	<0·0001	8·2 (5·5–12·0)	8·5 (5·4–12·2)	0·53	<0·0001
Total fat mass indexed for squared height	0·41 (0·28–0·60)	0·30 (0·20–0·49)	<0·0001	0·70 (0·55–0·90)	0·32 (0·21–0·52)	<0·0001	0·0036
Trunk fat mass indexed for squared height	0·16 (0·10–0·24)	0·10 (0·07–0·19)	<0·0001	0·30 (0·20–0·44)	0·27 (0·17–0·40)	0·012	0·53
Systolic blood pressure, mm Hg	104 (9)	103 (9)	0·0056	112 (8)	122 (9)	<0·0001	<0·0001
Diastolic blood pressure, mm Hg	60 (8)	59 (8)	<0·0001	65 (6)	63 (6)	<0·0001	0·45
PWV carotid-femoral, m/s	NA	NA	..	5·5 (0·6)	6·0 (0·7)	<0·0001	..
C-reactive protein, mg/L	0·26 (0·14–0·65)	0·15 (0·09–0·36)	<0·0001	0·66 (0·32–1·55)	0·44 (0·26–0·92)	<0·0001	0·056
Cholesterol, mmol/L	4·33 (0·62)	4·18 (0·65)	<0·0001	3·94 (0·68)	3·56 (0·62)	<0·0001	<0·0001
Triglycerides, mmol/L	1·04 (0·79–1·38)	0·97 (0·74–1·33)	0·0014	0·75 (0·6–0·98)	0·74 (0·59–0·95)	0·18	0·16
HDL, mmol/L	1·38 (0·30)	1·44 (0·31)	<0·00001	1·36 (0·32)	1·19 (0·26)	<0·0001	<0·0001
LDL, mmol/L	2·20 (0·57)	2·23 (0·58)	<0·0001	2·20 (0·63)	2·43 (0·55)	<0·0001	0·40
Insulin, pmol/L	NA	NA	..	43·6 (32·5–58·3)	36·1 (26·4–51·0)	<0·0001	..
Glucose, mmol/L	4·9 (4·7–5·0)	4·9 (4·8–5·1)	0·017	4·9 (4·7–5·1)	5·1 (4·9–5·4)	<0·0001	<0·0001
Adiponectin, × 10^3^ mg/mL	12·9 (9·4–16·9)	11·9 (8·9–15·5)	0·00020	NA	NA	..	..
Leptin, ng/mL	6·8 (4·2–11·5)	3·9 (2·6–7·3)	<0·0001	NA	NA	..	..

Data are mean (SD) or median (IQR). p values represents differences between sex at the respective timepoints. BMI=body-mass index. PWV=pulse wave velocity. NA=not available. HDL=high-density lipoprotein. LDL=low-density lipoprotein.

* p value for the difference in change from ages 9 years to 17 years between female and male participants.

**Table 2:** Study population characteristics according to normal or high fat mass at ages 9 years and 17 years

		**Age 9 years**			**Age 17 years**			p value for change*
		Normal FMI (n=2359)	High FMI (n=785)	p value	Normal FMI (n=2490)	High FMI (n=828)	p value	
Sex								
	Female	1278 (54·2%)	425 (54·1%)	..	1131 (45·4%)	376 (45·4%)	..	..
	Male	1081 (45·8%)	360 (45·9%)	..	1359 (54·6%)	452 (54·6%)	..	..
Weight, kg		35·5 (6·4)	44·3 (7·8)	<0·0001	64 (11·6)	72·5 (13·1)	<0·0001	<0·0001
Height, cm		141 (5)	152 (4)	<0·0001	169 (8)	180 (8)	<0·0001	0·81
BMI, kg/m^2^		17·7 (2·7)	19·0 (3·1)	<0·0001	22·4 (3·7)	22·4 (3·7)	0·91	0·0029
Waist-to-height ratio		0·44 (0·42–0·47)	0·44 (0·42–0·48)	0·83	0·44 (0·42–0·48)	0·44 (0·41–0·47)	0·0050	0·026
Total fat mass, kg		5·9 (4·3–8·0)	14·0 (12·6–16·8)	<0·0001	13·0 (8·6–17·3)	28·0 (24·8–34·1)	<0·0001	<0·0001
Trunk fat mass, kg		2·1 (1·5–3·0)	6·0 (4·9–7·5)	<0·0001	8·7 (5·7–12·5)	7·8 (4·7–10·8)	0·0096	<0·0001
Total fat mass indexed for squared height, kg/m^2^		0·29 (0·21–0·40)	0·72 (0·62–0·88)	<0·0001	0·45 (0·27–0·61)	0·99 (0·86–1·19)	<0·0001	<0·0001
Trunk fat mass indexed for squared height, kg/m^2^		0·10 (0·07–0·15)	0·31 (0·25–0·39)	<0·0001	0·29 (0·19–0·44)	0·27 (0·16–0·37)	0·025	<0·0001
Systolic blood pressure, mm Hg		103 (9)	105 (9)	<0·0001	116 (10)	118 (10)	<0·0001	0·00020
Diastolic blood pressure, mm Hg		59 (8)	59 (8)	0·22	64 (6)	64 (6)	0·64	0·0067
PWV femoral, m/s		NA	NA	..	5·7 (0·7)	5·9 (0·8)	<0·0001	..
C-reactive protein, mg/L		0·20 (0·11–0·53)	0·22 (0·11–0·49)	0·72	0·53 (0·29–1·3)	0·55 (0·27–1·20)	0·95	0·45
Cholesterol, mmol/L		4·29 (0·64)	4·17 (0·61)	0·00017	3·77 (0·67)	3·69 (0·70)	0·0073	0·72
Triglycerides, mmol/L		1·01 (0·78–1·35)	1·00 (0·75–1·39)	0·61	0·74 (0·60–0·96)	0·75 (0·60–0·97)	0·90	0·87
HDL, mmol/L		1·41 (0·31)	1·38 (0·29)	0·028	1·27 (0·31)	1·27 (0·30)	0·72	0·45
LDL, mmol/L		2·36 (0·58)	2·27 (0·55)	0·0019	2·12 (0·61)	2·05 (0·61)	0·019	0·90
Insulin, pmol/L		NA	NA	..	39·8 (29·9–55·6)	39·7 (28·9–54·7)	0·59	..
Glucose, mmol/L		4·9 (4·7–5·1)	4·9 (4·8–5·1)	0·65	5·0 (4·8–5·2)	5·0 (4·8–5·3)	0·80	0·19
Adiponectin, × 10^3^ mg/mL		12·0 (9·2–16·3)	12·0 (8·9–16·1)	0·30	NA	NA	..	..
Leptin, ng/mL		4·7 (3·0–8·8)	7·0 (4·2–13·3)	<0·0001	NA	NA	..	..

Data are mean (SD) or median (IQR). Normal fat mass (≤75th percentile) and high fat mass (>75th percentile) groups for each time period were defined according to the distribution of total fat mass at ages 9 years and 17 years after adjustment for height and sex. Fat measurements (total and trunk fat mass) are provided on the basis of unadjusted groups due to further height indexing. p values represent differences between high and normal fat mass at the respective timepoints. FMI=fat mass index. BMI=body-mass index. PWV=pulse wave velocity. HDL=high-density lipoprotein. LDL=low-density lipoprotein. NA=not available.

* p value for the difference in change from ages 9 years to 17 years between participants with normal fat mass and high fat mass.

In the longitudinal analysis, participants with sufficient measurements were classified into high, middle, and low trajectories of total and trunk FMI created by LCG analysis according to longitudinal fat mass. Regarding total FMI, most of the 3417 children followed the middle trajectory (n=1544), while 560 participants followed the high trajectory and 1313 participants followed the low trajectory (appendix). Three trunk FMI groups were identified on 3290 participants (appendix): participants with high truncal FMI throughout adolescence (n=569), the middle trajectory (n=991), and low trajectory (n=1730). Finally, we identified two trajectories of fat-free mass index (low [n=1888] and high [n=1529]) and three trajectories of BMI changes (low [n=1222], middle [n=1522], and high [n=679]).

In cross-sectional analyses at age 17 years, total fat mass was positively associated with PWV and there was weak evidence of a positive association between trunk fat mass and PWV (table 3). These associations remained following adjustment for demographic, haemodynamic, and metabolic parameters (table 3). After multiple imputation, total fat mass was still independently positively associated to PWV (0·01 m/s per kg, 95% CI 0·00–0·02; p=0·0056). Following cross-sectional analysis, only fat mass at ages 9, 11, and 17 years were independently associated with PWV (appendix) and the strength of association was highest for 9 years of age, suggesting that this might be an important age at which to test whether weight loss intervention could have a long-term beneficial effect on arterial stiffness (appendix).

**Table 3:** Cross-sectional associations of adiposity measures with PWV at age 17 years

	Univariate analysis (n=3333)*		**Multivariable adjusted analysis (n=1910)**†	
	PWV coefficient, m/s (95% CI)	p value	PWV coefficient, m/s (95% CI)	p value
Total fat mass, kg	0·004 (0·001 to 0·006)	0·0081	0·010 (0·002 to 0·016)	0·016
Trunk fat mass, kg	0·007 (−0·001 to 0·020)	0·073	0·010 (−0·010 to 0·024)	0·25
BMI, kg/m^2^	0·04 (−0·00 to 0·10)	0·23	..	..
Waist-to-height ratio	0·20 (−0·34 to 0·73)	0·48	..	..

Multivariable adjusted analysis was done for significant univariate analyses. PWV=pulse wave velocity. LDL=low-density lipoprotein. BMI=body-mass index.

* Adjusted for sex.

† Adjusted for sex, systolic blood pressure, LDL, C-reactive protein, socioeconomic status, Z score of BMI, and birthweight (or fat when BMI was used as a dependent variable).

Persistently high total FMI was associated with greater PWV at age 17 years (table 4; appendix). This association remained after multivariable adjustment. No interaction was found between sex and FMI on PWV at age 17 years (data not shown). After multiple imputation for missing values, the association between persistently high total FMI and greater PWV remained (0·10 m/s per kg/m^2^, 95% CI 0·03–0·18; p=0·0069). Similarly, persistently high trunk FMI was associated with greater PWV and retained its association after multivariable adjustment (table 4) and imputation (0·12 m/s per kg/m^2^, 0·04–0·19, p=0·0017).

**Table 4:** Associations of adiposity trajectories through adolescence with PWV

	Univariate analysis (n=3417)*		**Multivariable adjusted analysis (n=1940)**†	
	PWV coefficient, m/s (95% CI)	p value	PWV coefficient, m/s (95% CI)	p value
**Total FMI (kg/m^2^)**				
Low trajectory	−0·00 (−0·06 to 0·05)	0·95	0·02 (−0·06 to 0·09)	0·65
High trajectory	0·07 (0·01 to 0·14)	0·027	0·15 (0·05 to 0·24)	0·0044
**Trunk FMI (kg/m^2^)**				
Low trajectory	−0·03 (−0·08 to 0·02)	0·27	−0·03 (−0·10 to 0·04)	0·41
High trajectory	0·10 (0·03 to 0·17)	0·0041	0·15 (0·06 to 0·25)	0·0021
**BMI trajectories (kg/m^2^)**				
Low trajectory	0·01 (−0·04 to 0·06)	0·59	−0·05 (−0·11 to 0·02)	0·18
High trajectory	0·09 (0·03 to 0·16)	0·0030	0·04 (−0·04 to 0·13)	0·34

In each case, the middle trajectory is the reference group. PWV=pulse wave velocity. LDL=low density lipoprotein. FMI=fat mass indexed to squared height. BMI=body-mass index.

* Adjusted for sex.

† Adjusted for sex, systolic blood pressure, LDL, C-reactive protein, socioeconomic status, *Z*-score of BMI at 17 years (except for models incorporating trajectories of BMI), and birthweight.

We found little evidence to support the association of high fat-free mass index and PWV at age 17 years compared to low fat-free mass index (data not shown).

Compared with participants who were metabolically healthy and had normal FMI, participants with persistently high FMI had elevated PWV (6·2 m/s [95% CI 5·9–6·4] for metabolically unhealthy participants and 6·0 m/s [5·9–6·0] for metabolically healthy participants), as did children who simultaneously increased their FMI from ages 9 to 17 years and were metabolically unhealthy (PWV 6·2 m/s, 95% CI 5·9–6·5; figure). Notably, participants who decreased their FMI to normal ranges from age 9 years to age 17 years had comparable PWV to those who had normal FMI throughout those ages irrespective of their metabolic status (5·8 m/s [5·7–5·9] for metabolically healthy participants and 5·9 m/s [5·6–6·1] for metabolically unhealthy participants). Participants who had normal FMI at both periods but were metabolically unhealthy showed increased PWV compared to normal FMI and metabolic healthy group (PWV 5·7 m/s [5·7–5·8] and 5·9 m/s [5·8–6·0] for metabolically healthy and unhealthy, respectively). Overall, the combination of high total fat mass and a metabolically unhealthy profile was linearly associated with increased PWV at age 17 years: a mean expected adjusted increase of 0·08 m/s (95% CI 0·03–0·13) was shown in PWV per ascending category of combined fat mass exposure and metabolic health (p for linear trend=0·0034), with the lowest category comprising children who had normal fat mass and were metabolically healthy across adolescence and the highest comprising children who were obese and metabolically unhealthy at both ages 9 and 17 years (figure). After imputation, this effect did not change (mean increase 0·03 m/s [0·01–0·04] per ascending category; p<0·0001).

**Figure:**
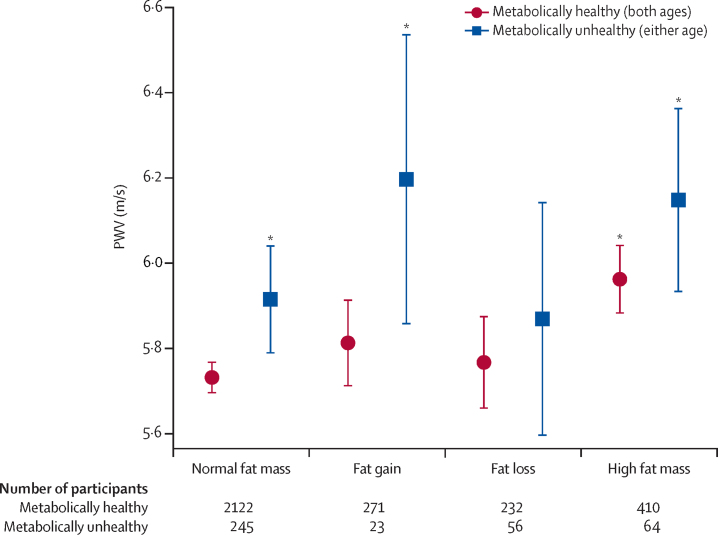
PWV per combinations of metabolic health and adiposity status at 9 and 17 years of age Metabolically healthy includes all participants categorised as metabolically healthy at both 9 and 17 years of age, whereas metabolically unhealthy includes all participants categorised as metabolically unhealthy at either 9 or 17 years of age. The reference category comprised children who had normal fat mass and were metabolically healthy across adolescence. p values for linear trend are derived from multivariable regression analysis for PWV at 17 years adjusted for sex, height, socioeconomic status, systolic blood pressure, C-reactive protein, LDL, Z score of BMI, and birthweight. BMI=body-mass index. LDL=low-density lipoprotein. PWV=pulse wave velocity. *Significant difference (p<0·05) from the reference category.

BMI Z score correlated with total FMI at age 17 years (*r*_s_ = 0·76; p<0·0001) but not at age 9 years (*r*_s_ = −0·006; p=0·84). At age 17 years, 1090 (33%) of 3318 participants were misclassified in their adiposity status by BMI compared with total FMI (ie, participants were either considered to be normal weight by BMI but overweight or obese by total FMI or vice versa). Waist-to-height ratio was not associated with total or trunk FMI at any timepoint during adolescence. BMI and waist-to-height ratio were not related to arterial stiffness (table 3). High trajectory of BMI across adolescence was related to greater arterial stiffness but adjustment for cardiometabolic factors, including birthweight, attenuated the association with PWV (table 4; appendix).

## Discussion

In this study, we have shown that persistent high fat mass during adolescence is independently associated with increased arterial stiffness, one of the earliest markers of atherosclerotic disease. This adverse association was not apparent when routine clinical anthropometric measures of adiposity such as BMI or waist-to-height ratio were used and was amplified in the presence of a metabolically unhealthy phenotype. We also identified adolescence as an important period for preventive interventions because participants who lost fat during adolescence had similar arterial stiffness to those who had normal fat mass throughout. These findings suggest that targeted interventions to reduce obesity and its consequences in adolescence might translate into long-term vascular benefit.

Obesity in the young remains a highly complex medical problem in terms of cause. Several longitudinal studies have shown that obesity tracks from childhood to adulthood and increased weight gain in early childhood can predict obesity in adolescence and might help to identify individuals at risk.^15^ Although obesity has been associated with adverse cardiovascular outcomes in adulthood, its influence during the long preclinical phase of arterial disease remains less well defined. Adaptive vascular responses in peripheral arteries in relation to increased adiposity in childhood has previously been shown.^5^, ^6^, ^7^ Because evidence suggests that central arteries (ie, the aorta) might be more vulnerable to atherosclerosis, we chose to assess aortic stiffness as measured by PWV between carotid to femoral arterial segments^16^ rather than carotid to radial arterial segments, which reflect changes in a muscular artery (the brachial artery) that is not reflective of developing atherosclerosis. Studies have shown that PWV measurements are reproducible and that increased PWV can predict adverse outcome such as cardiovascular events and mortality in adults.^16^ The present study has shown that increases in directly measured body fat, as assessed by DEXA during adolescence, appear to be associated with greater PWV in late adolescence. These findings contradict a previous study, which showed that increased adiposity is associated with reduced arterial stiffness in children and adolescents and that an adverse association is only apparent in later life.^17^ It is possible that the use of body fat percentage rather than absolute values of fat mass could account for this discrepancy.

The pattern of fat distribution, rather the quantity of fat mass, might also be important in determining cardiovascular risk, as excess fat in the central regions of the body rather than the gluteofemoral area has been more strongly associated with deleterious health outcomes.^18^ In our study, although truncal fat was associated with increased arterial stiffness, only total fat was related to PWV in multivariable analysis. As total fat measured by DEXA provides information of both visceral and subcutaneous fat, this finding suggests that any type of fat might be important in determining vascular risk at this stage, whereas metabolic risk is more closely related to BMI and truncal fat mass.^19^ Previous studies have shown a strong correlation between truncal and visceral fat in adults;^20^ however, such a link is not clear in adolescence. Although waist circumference provides a reliable estimate of visceral adipose tissue in some studies,^21^ this finding is not supported in others.^22^ In our study, the association between waist-to-height ratio and DEXA-derived obesity measurements was weak for total and truncal fat, suggesting that the contribution of visceral fat might be less important at this age. It is also possible that the amount of visceral fat in this generally healthy cohort of young participants is small and therefore we were unable to show significant associations.

Adolescents affected by obesity are also at increased risk of developing other cardiovascular risk factors such as dyslipidaemia, hypertension, and insulin resistance—a combination commonly referred to as metabolic syndrome.^13^, ^23^ Whether a constellation of these risk factors increases cardiovascular risk over and above adiposity in children and adolescents remains a matter of controversy. Some, but not all, studies have shown greater alterations of cardiovascular structure and function in children with the combination of obesity and metabolic syndrome compared with children without it.^23^ In the ALSPAC cohort, the presence of established metabolic syndrome as defined in adults is low, and we have used percentiles to separate the metabolically healthy from unhealthy participants, as previously used by others.^13^ We used a combination of three or more cardiovascular risk factors to define metabolic health and, consistent with previous reports, we showed that participants who had the persistent high fat mass and had a metabolically unhealthy phenotype had the highest arterial stiffness. These findings suggest that risk factor management is also important in reducing cardiovascular risk and complement previous reports showing that the number of cardiovascular events could be decreased by treatment of obesity-related risk factors such as hypertension and dyslipidaemia.^24^ In the current study, participants who had high fat mass but were yet to accrue metabolic abnormalities still showed elevated arterial stiffness, suggesting that even in the absence of additional risk factors, strategies to reduce fat mass could be beneficial. In the 1946 birth cohort, our group has previously shown that achieving weight loss at any point in life, even if not sustained, was beneficial for vascular health.^25^ Consistent with this and other observations, ^26^ we show in this study that reduction of fat mass in adolescence is beneficial because participants who normalised their FMI in adolescence had comparable arterial stiffness to those who had consistently normal FMI. The association of total fat mass with arterial stiffness was stronger at 9 years of age than the other time periods, which suggests that strategies to prevent obesity in this period could be beneficial for future cardiovascular health; however, this needs further study.

In addition to DEXA, we also measured other more commonly used adiposity indices and related them to fat mass and arterial stiffness. As previously described, the association between BMI and body fat is weak in children and strong association becomes more apparent only in adulthood.^27^ This probably reflects the inability of BMI to discriminate between fat and fat-free mass and to decipher physiological changes that alter their relative amounts during adolescence with growth and puberty. In adolescence, the differentiation between growth and increasing adiposity becomes more challenging, partly because of rapid changes in body composition in response to growth and sex hormones. In this cohort, BMI was not associated with arterial stiffness in the cross-sectional analyses but the relationship was apparent in the longitudinal analysis and was mostly explained by the presence of metabolic abnormalities. These results suggest that in adolescence, a more detailed phenotype such as DEXA-derived FMI might be needed when assessing a relationship between adiposity and certain markers of vascular disease. As a measure of combined risk from adiposity and metabolic health, BMI could still be considered a useful tool for risk stratification in the clinical setting.

Our study has a number of strengths and limitations. A variety of metabolic parameters were collected in childhood and adolescence, which allowed us to identify the combined effect of adiposity status and metabolic profile on arterial stiffness. However, the presence of unmeasured or residual confounders cannot be excluded. Indeed, several studies have shown strong associations between leptin, adiponectin, and cardiovascular disease.^28^ In our study, adipocytokines were only measured in childhood; thus, we were unable to determine whether changes in adipocytokine levels and other unmeasured factors can account for the noted associations. In addition, the vast majority of our participants were of white British background, so extrapolation to other ethnic backgrounds is inappropriate. Non-fasting blood samples were collected in childhood and fasting blood samples were collected at 17 years of age. Although lipid profile might not be affected by fasting state,^29^ some variability in the measurements for triglycerides and glucose, which might have led to misclassification of the metabolic profile of participants, cannot be excluded. However, we elected to use accelerometers at two timepoints to accurately assess fitness levels rather than relying on self-reported questionnaires, which should increase the accuracy of our results. Finally, as in all cohort studies, causal associations cannot be established. Despite these limitations, our serial assessment allowed us to determine the relationship between fat mass and that of metabolic parameters on arterial stiffness.

In conclusion, we have shown that persistent high fat mass from childhood was associated with an adverse effect on arterial stiffness at age 17 years and this effect was apparent in the presence or absence of metabolic abnormalities. Individuals who normalised their fat mass during adolescence had comparable PWV to those who had normal fat mass throughout. These findings suggest further interventional studies are needed to assess whether aggressive weight loss and metabolic control strategies during adolescence could provide long-term vascular benefits.
