# Supplementary material for: Association between fat mass through adolescence and arterial stiffness: a population-based study from The Avon Longitudinal Study of Parents and Children
Source: Lancet Child Adolesc Health. 2019 Jul;3(7):474–81. doi: 10.1016/S2352-4642(19)30105-1 (PMC6558973; doi:10.1016/S2352-4642(19)30105-1)
Supplement: Supplementary appendix [file mmc1.pdf]

THE LANCET
Child & Adolescent Health

Supplementary appendix

This appendix formed part of the original submission and has been peer reviewed. We post it as supplied by the authors.

Supplement to: Dangardt F, Charakida M, Georgiopoulos G, et al. Association between fat mass through adolescence and arterial stiffness: a population-based study from The Avon Longitudinal Study of Parents and Children. *Lancet Child Adolesc Health* 2019; published online May 20. http://dx.doi.org/10.1016/S2352-4642(19)30105-1.

Table of Contents

Dangardt et al 1

1.1 Adiposity characterisation 3

1.2 Measurement of cardiovascular risk factors 3

1.3 Statistical methods 4

Latent Class Growth Mixture Modelling 5

Supplementary Table 1. 6

Supplementary Table 2. 7

Supplementary Table 3. 8

Supplementary Table 4. 9

Cross-sectional associations of total fat measurements across different ages (9 to 17 years) with PWV (m/s) at 17 years 9

Figure Legends 10

References 11

1.1 Adiposity characterisation

Weight was measured to the nearest 0·1kg using Tanita scales (Tanita Europe BV, Amsterdam, Netherlands) at two yearly intervals from 9 to 17 years. Height was measured to the nearest 0·1 cm using a Harpenden stadiometer (Holtain Ltd, Crymych Pembrokeshire, UK). BMI was calculated as weight/height^2 (weight in kg and height in meters) at each period. Waist circumference was measured using a flexible tape, to the nearest 1 mm, at the midpoint between the lower ribs and the iliac crest and the ratio of waist circumference to height was calculated. [1] Measurements were collected at two-yearly intervals from age 9 to 17 years.

Fat mass was assessed using DEXA at 9, 11, 13, 15 and 17 years. A Lunar Prodigy narrow fan-beam densitometer (GE Healthcare, Bedford, UK) was used to perform a whole-body DEXA scan, where total fat mass and trunk fat mass were measured. Truncal fat mass was estimated using the automatic region of interest above the pelvis that included chest and abdomen. Total/trunk fat mass indices (FMI) were calculated by dividing total/trunk fat mass by height^2 and fat-free mass index (FFMI) was generated by dividing fat free mass by height^2. Participants were classified as "high FMI" if total sex-and height-adjusted FMI >75th centile.

1.2 Measurement of cardiovascular risk factors

Blood pressure at age 9 and 17 years was recorded in the right arm in the seated position at 9 and supine at 17 using a Dinamap 9301 Vital Signs Monitor (Morton Medical, London, UK). Blood pressure measurements were performed at the time of carotid to femoral PWV assessment at 17 years. The mean of two blood pressure values were used for analysis. The size of the cuff was selected according to the upper arm circumference.

Non-fasting blood samples were taken using standard procedures at 9 years, whereas overnight fasting samples were collected for analysis at 17 years. Plasma lipids (total cholesterol, triglycerides, and high-density lipoprotein (HDL) cholesterol) were measured by modification of the standard Lipid Research Clinics Protocol using enzymatic reagents for lipid determination. All assay coefficients of variation were <5%. Non-HDL cholesterol was calculated as total cholesterol minus HDL cholesterol. The amount of weekly vigorous physical activity was assessed by accelerometer (Actigraph, Pensacola, Florida, USA) at 9 and 15 years of age. Similarly to the modified NCEP and IDS definitions of paediatric metabolic syndrome[2], subjects were classified as "metabolically unhealthy" at either 9 or 17 years if they had 3 or more of the following risk factors: systolic blood pressure (SBP) >75th centile, HDL<25th centile, triglycerides >75th centile and glucose >75th centile.[2]

1.3 Statistical methods

Multivariable models were built on the basis of *a priori* variable selection, according to previous medical literature with adjustment for established CV risk factors[3] and exposure to variables previously associated with increased PWV.[4] An interaction effect between sex and FMI categories or combined adiposity and metabolic health status was tested on PWV at 17 years by implementing likelihood ratio tests.

In multivariable regression models, we replaced missing data for variables of interest by multiple imputation using the Markov chain Monte Carlo (MCMC) method. We imputed 20 datasets in total under the missing at random assumption for all missing values through chained equations. We used 10 iterations for the burn-in period. Imputed variables included total and trunk fat mass at 17 years, parental and adolescent's smoking, birthweight, puberty status (Tanner stage), physical activity at 15 years, SES, SBP, LDL, BMI and CRP at 17 years (expanded multivariable

model). Additional imputations were performed for 9- and 17-years' HDL, triglycerides, fasting glycose and high total fat mass at 9 years. Statistical analysis was performed by Stata package, version 13.1 (StataCorp, College Station, Texas USA). The "lcmm" package in RStudio (version 1.1.414) was used in Latent Class Growth Mixture Modelling analysis (LCGMM) analysis. All tests were two-sided. A priori, we planned to draw conclusions based on effect estimates and their CIs, rather than statistical tests using an arbitrary P value cut-off. For example, given two effects with the same point estimate—one with narrow CIs, the other with wider CIs that may even include the null—we describe both as showing the same effect but note that one is more imprecisely estimated and should be treated with more caution until replicated in a larger sample.

Latent Class Growth Mixture Modelling

To assess the effect of longitudinal changes in adiposity indices through adolescence on arterial stiffness, we performed Latent Class Growth Mixture Modelling analysis (LCGMM). By LCGMM, we identified distinct patterns of total fat mass, trunk fat mass, lean mass and BMI changes from age 9 to 17 years old in our cohort. Multiple measurements of total fat, trunk fat and lean mass were indexed for height changes (i.e. height squared) through adolescence by dividing each observation with squared height. Latent Class Growth models contained random intercept and slope(s) variance to account for between-subjects heterogeneity in longitudinal changes in obesity measurements while linear, quadratic and cubic specifications for the within-subject response of these variables as a function of increasing age were evaluated[5]. One (all subjects are assumed to follow the same underlying trajectory over time) to five classes were tested in LCGMM models. To enhance numeric stability and attenuate differences between genders, z-scores for responses across time were used.

Supplementary Table 1.

Latent Class Growth Mixture Modelling: selection of optimal number of classes for trajectories of total FMI changes across the period of 9 to 17 years old. Polynomial specification describes the within-subject functional association of longitudinal total FMI changes with increasing age and includes linear, quadratic and cubic form to allow for curvilinear patterns. BIC, Bayes information criterion; FMI, fat mass indexed to height^2; n.a, non applicable; NC, non-converged models; NP, not provided due to suboptimal class separation (empty or 100% containing latent classes). The model selected for trajectories of total FMI changes is highlighted in bold text.

MODEL	NB. LATENT **Classes**	POLYNOMIAL **Degree**	LOG-LIKELIHOOD	**Bic**	% PARTICIPANTS **Per Class**	MEAN **Posterior Probabilities**	POSTERIOR **Probabilities** > 0·7 (%)	CONVERGENCE	**Class Separation**
**1**	1	Linear	-11952·05	23952·91	100	(n.a)	(n.a)	Yes	n.a
**2**		Quadratic	-11660·37	23402·11	100	(n.a)	(n.a)	Yes	n.a
**3**		Cubic	-10539·72	21201·49	100	(n.a)	(n.a)	Yes	n.a
**4**	2	Linear	-11742·25	23565·86	(23·32/76·68)	(0·8219/0·8346)	(71·89/86·07)	Yes	Yes
**5**		Quadratic	-11305·6	22733·24	(70·24/29·76)	(0·8463/0·8552)	(86·58/77·58)	Yes	Yes
**6**		Cubic	NC	NC	NC	NC	NC	No	n.a
**7**	3	Linear	-11696·75	23507·41	(55·31/11·18/33·51)	(0·8482/0·8331/0·6879)	(80·85/73·3/48·3)	Yes	Yes
8		**Quadratic**	**-11169·37**	**22501·48**	**(38·43/45·19/16·39)**	**(0·8167/0·7552/0·8468)**	**(78·22/65·87/78·04)**	**Yes**	**Yes**
**9**		Cubic	NC	NC	NC	NC	NC	No	n.a
**10**	4	Linear	-11771·46	23664·98	(0/0/86·04/13·96)	NP	NP	Yes	No
**11**		Quadratic	NC	NC	NC	NC	NC	No	n.a
**12**		Cubic	NC	NC	NC	NC	NC	No	n.a
**13**	5	Linear	-11771·46	23689·39	(0/0/0/85·13/14·87)	NP	NP	Yes	No
**14**		Quadratic	NC	NC	NC	NC	NC	No	n.a
**15**		Cubic	NC	NC	NC	NC	NC	No	n.a

Supplementary Table 2.

Latent Class Growth Mixture Modelling: selection of optimal number of classes for trajectories of trunk FMI changes across the period of 9 to 17 years old. Polynomial specification describes the within-subject functional association of longitudinal trunk FMI changes with increasing age and includes linear, quadratic and cubic form to allow for curvilinear patterns. BIC, Bayes information criterion; FMI, fat mass indexed to height^2; n.a, non-applicable; NC, non-converged models; NP, not provided due to suboptimal class separation (empty or 100% containing latent classes). The model selected for trajectories of trunk FMI changes is highlighted in bold text.

MODEL	NB. LATENT **Classes**	POLYNOMIAL **Degree**	LOG-LIKELIHOOD	**Bic**	% PARTICIPANTS **Per Class**	MEAN **Posterior Probabilities**	POSTERIOR **Probabilities** > 0·7 (%)	CONVERGENCE	**Class Separation**
**1**	1	Linear	-7948·4	15945·4	-100	(n.a)	(n.a)	Yes	n.a
**2**		Quadratic	-7935·74	15952·47	-100	(n.a)	(n.a)	Yes	n.a
**3**		Cubic	-7616·18	15353·84	-100	(n.a)	(n.a)	Yes	n.a
**4**	2	Linear	-7450·83	14982·66	(26·29/73·71)	(0·8602/0·8737)	(78·84/89·98)	Yes	Yes
**5**		Quadratic	-7428·98	14979·43	(73·13/26·87)	(0·8737/0·8649)	(90·07/79·52)	Yes	Yes
**6**		Cubic	NC	NC	NC	NC	NC	No	n.a
**7**	3	Linear	-7380·81	14874·99	(53·83/17·9/28·27)	(0·7194/0·8317/0·625)	(56·92/74·02/28·06)	Yes	Yes
8		**Quadratic**	**-7350·62**	**14863·2**	**(30·12/52·58/17·29)**	**(0·6437/0·7325/0·8344)**	**(29·26/60·69/74·69)**	**Yes**	**Yes**
**9**		Cubic	NC	NC	NC	NC	NC	No	n.a
**10**	4	Linear	-7621·33	15364·14	(83·71/0/0/16·29)	NP	NP	Yes	No
**11**		Quadratic	-7609·48	15397·12	(0/0/84·95/15·05)	NP	NP	Yes	No
**12**		Cubic	NC	NC	NC	NC	NC	No	n.a
**13**	5	Linear	NC	NC	NC	NC	NC	No	n.a
**14**		Quadratic	NC	NC	NC	NC	NC	No	n.a
**15**		Cubic	NC	NC	NC	NC	NC	No	n.a

Supplementary Table 3.

Latent Class Growth Mixture Modelling: selection of optimal number of classes for trajectories of BMI changes across the period of 9 to 17 years old. Polynomial specification describes the within-subject functional association of longitudinal fat mass changes with increasing age and includes linear, quadratic and cubic form to allow for curvilinear patterns. BIC, Bayes information criterion; BMI, body mass index, n.a, non-applicable; NC, non-converged models; NP, not provided due to suboptimal class separation (empty or 100% containing latent classes. The model selected for trajectories of BMI changes is highlighted in bold text.

MODEL	NB.LATENT Classes	POLYNOMIAL Degree	LOG-Likelihood	Bic	% PARTICIPANTS Per Class	MEAN Posterior Probabilities	POSTERIOR Probabilities > 0·7 (%)	CONVERGENCE	Class Separation
**1**	1	Linear	-13472·6	26994·03	-100	(n.a)	(n.a)	Yes	n.a
**2**		Quadratic	-12676·35	25434·08	-100	(n.a)	(n.a)	Yes	n.a
**3**		Cubic	-11814·95	23751·97	-100	(n.a)	(n.a)	Yes	n.a
**4**	2	Linear	-12964·87	26011·13	(76·99/23·01)	(0·8644/0·8639)	(90·4/80·33)	Yes	Yes
**5**		Quadratic	-11883·2	23888·48	(74·65/25·35)	(0·8901/0·8838)	(91·59/83·41)	Yes	Yes
**6**		Cubic	-10796·64	21764·2	(71·9/28·1)	(0·8935/0·9037)	(90·9/86·8)	Yes	Yes
**7**	3	Linear	-12882·66	25879·26	(38·38/43·4/18·22)	(0·7765/0·7306/0·8509)	(70·85/62·99/75·48)	Yes	Yes
**8**		Quadratic	-11748·56	23659·89	(39·14/43·98/16·88)	(0·7911/0·7638/0·8691)	(73·28/67·86/79·24)	Yes	Yes
9		**Cubic**	**-10627·07**	**21473·88**	**(35·69/44·48/19·83)**	**(0·7907/0·7746/0·8759)**	**(73·49/70·65/81·44)**	**Yes**	**Yes**
**10**	4	Linear	-13199·79	26521·67	(0/0/90·65/9·35)	NP	NP	Yes	No
**11**		Quadratic	-12390·02	24959·1	(90·68/0/0/9·32)	NP	NP	Yes	No
**12**		Cubic	-11525·6	23295·36	(90·8/0/0/9·2)	NP	NP	Yes	No
**13**	5	Linear	-13199·79	26546·08	(0/0/89·69/0/10·31)	NP	NP	Yes	No
**14**		Quadratic	-12390·02	24991·65	(0/89·54/0/0/10·46)	NP	NP	Yes	No
**15**		Cubic	NC	NC	NC	NC	NC	No	n.a

Supplementary Table 4.

Cross-sectional associations of total fat measurements across different ages (9 to 17 years) with PWV (m/s) at 17 years

	*Univariate association		**Multi-adjusted association	
	Coefficient 95% CI	P-value	Coefficient 95% CI	P-value
**Total fat mass at 9 (kg)**	0·01 (0·01 /0·02)	<0·0001	0·02 § (0·003/0·03)	<0·0001
**Total fat mass at 11 (kg)**	0·01 (0·01/0·013)	<0·0001	0·01 (0·001/0·02)	0·034
**Total fat mass at 13 (kg)**	0·01 (0·004-0·01)	0·035	0·02 (-0·01/0·01)	0·67
**Total fat mass at 15 (kg)**	0·004 (-0·001/0·01)	0·11	NP	NP
**Total fat mass at 17 (kg)**	0·004 (0·001/0·006)	0·0081	0·01 (0·002/0·016)	0·016

*Adjusted for sex
** Adjusted for sex, SBP, LDL, CRP, SES, height (for previous measurements of total fat mass), z-score of BMI and birth weight
§ Adjusted coefficients of total fat mass at 9 and 11 years were compared on the basis of bootstrapping with 1,000 replicates (9 years: mean adjusted increase 0·02, 95% CI 0·001/0·022 versus 11 years: 0·01 m/s, 95% CIs 0·001/0·01, p<0·001)
§ Adjusted coefficients of total fat mass at 9 and 17 years were compared on the basis of bootstrapping with 1,000 replicates (9 years: mean adjusted increase 0·02, 95% CI 0·001/0·02 versus 17 years: 0·01 m/s, 95% CIs 0·006/0·01, p<0·001)
Abbreviations: SBP, systolic blood pressure; LDL, low density lipoprotein; CRP, C-reactive protein; SES, socio-economic status; FMI; fat mass indexed to height2; BMI, body mass index; CI, confidence intervals; NP, not performed

Figure Legends

Supplementary Figure 1. Trajectories of a) total FMI b) trunk FMI and c) BMI changes across ages 9 to 17 years. Grey shading denotes confidence intervals. Solid, dashed and dotted lines represent low, intermediate and high exposure to adiposity. Trajectories have been derived from latent growth curve analysis, as described in detail in the Statistical Methods. A quadratic model with 3 latent classes was used to estimate trajectories of total and trunk FMI whereas a cubic model with 3 latent classes was chosen for BMI changes. Shaded areas indicate confidence intervals around each trajectory. FMI, fat mass indexed to height^2; BMI, body mass index

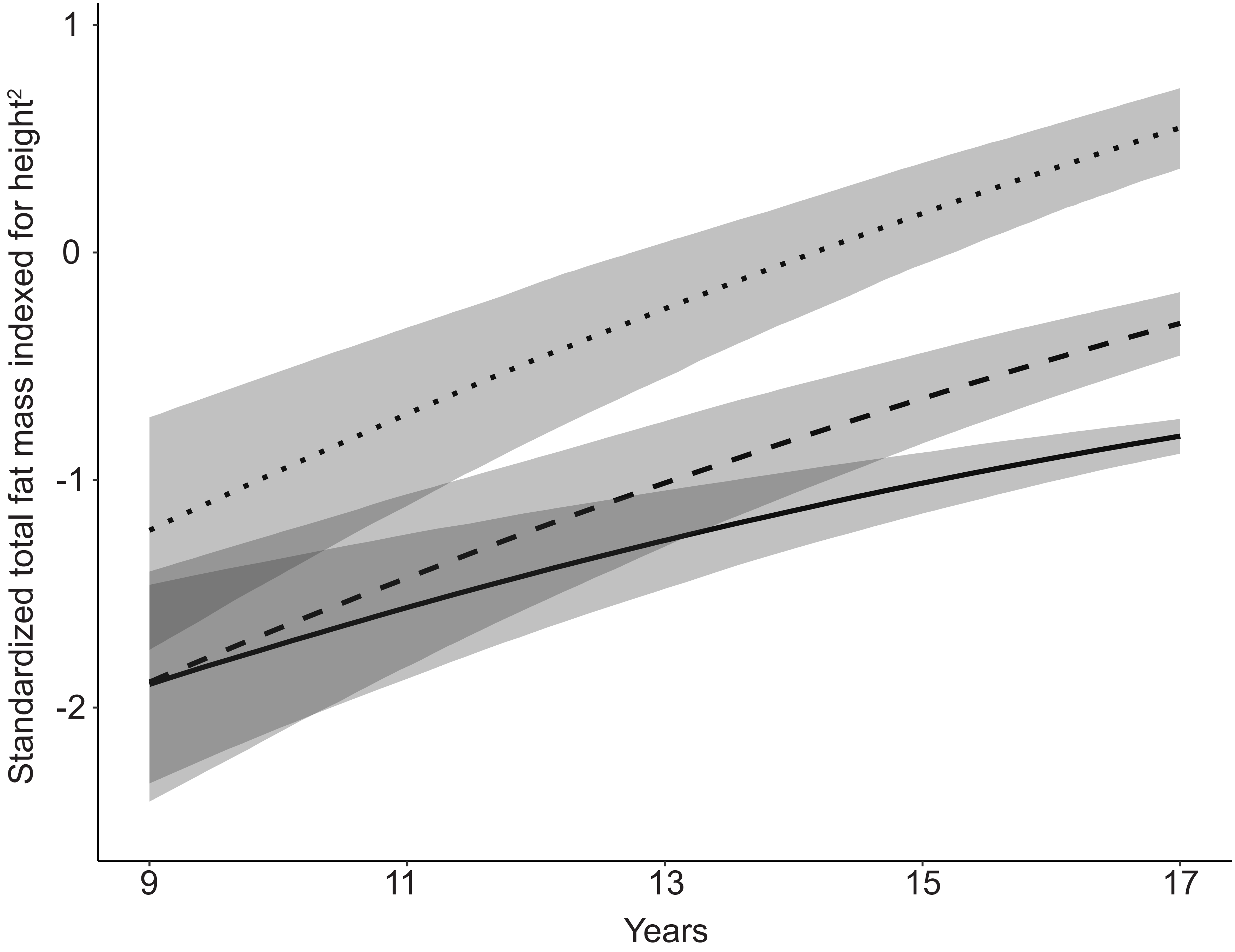

Standardized total fat mass indexed for height²
1
0
-1
-2
9
11
13
15
17
Years

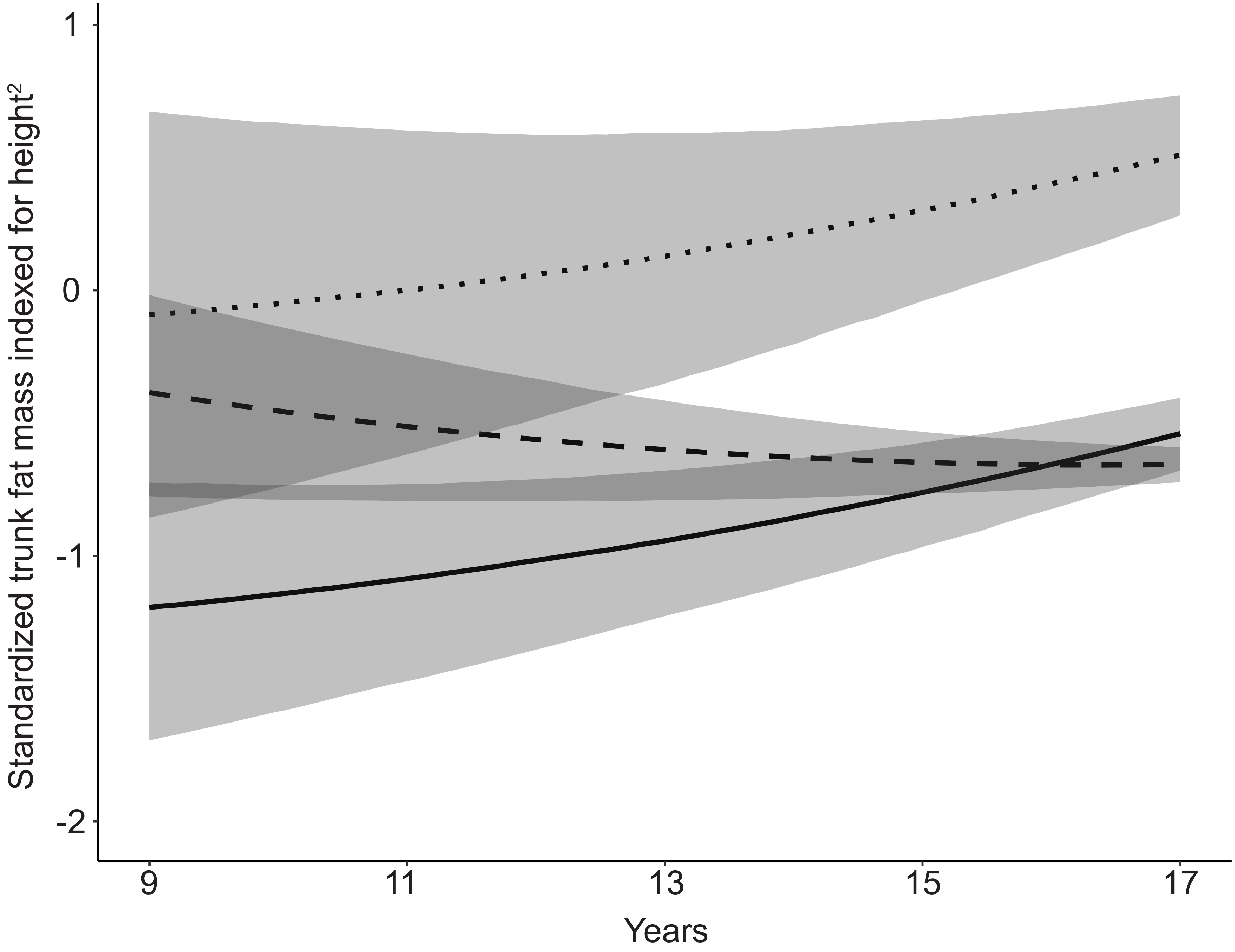

Standardized trunk fat mass indexed for height^2
1
0
-1
-2
9
11
13
15
17
Years

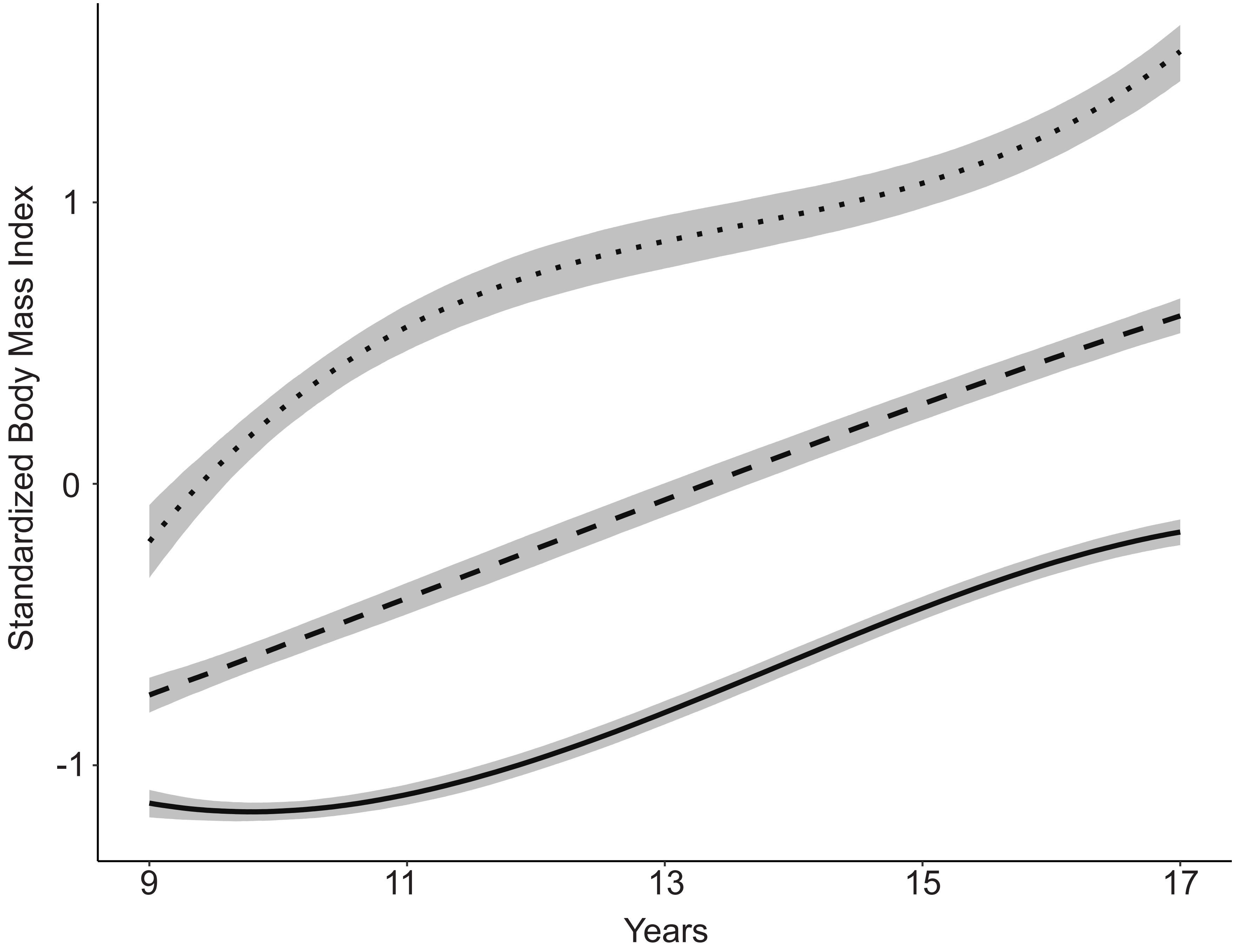
Standardized Body Mass Index
1
0
-1
9
11
13
15
17
Years

References

1. Charakida M, Jones A, Falaschetti E, et al. Childhood obesity and vascular phenotypes: a population study. *Journal of the American College of Cardiology* 2012; **60**(25): 2643-50.
2. Magnussen CG, Koskinen J, Chen W, et al. Pediatric Metabolic Syndrome Predicts Adulthood Metabolic Syndrome, Subclinical Atherosclerosis, and Type 2 Diabetes Mellitus but Is No Better Than Body Mass Index Alone. *Circulation* 2010; **122**(16): 1604-11.
3. The Emerging Risk Factors C. Separate and combined associations of body-mass index and abdominal adiposity with cardiovascular disease: collaborative analysis of 58 prospective studies. *The Lancet* 2011; **377**(9771): 1085-95.
4. Aatola H, Hutri-Kahonen N, Juonala M, et al. Lifetime risk factors and arterial pulse wave velocity in adulthood: the cardiovascular risk in young Finns study. *Hypertension* 2010; **55**(3): 806-11.
5. Buscot M-J, Thomson RJ, Juonala M, et al. Distinct child-to-adult body mass index trajectories are associated with different levels of adult cardiometabolic risk. *European Heart Journal* 2018; **39**(24): 2263-70.
